# Lactate and panic across infusion and exercise contexts: an interoceptive context-gating hypothesis

**DOI:** 10.3389/fpsyt.2026.1975318

**Published:** 2026-10-29

**Authors:** Xinze Li, Shikun Wang, Weifu Chen

**Affiliations:** 1 Qilu Institute of Technology, Jinan, Shandong, China; 2 Qufu Normal University, Qufu, Shandong, China; 3 Beijing Union University, Beijing, China

**Keywords:** acid–base regulation, exercise, insular cortex, interoception, lactate, panic disorder, panic provocation, predictive coding

## Abstract

Lactate is an oxidative fuel, a gluconeogenic precursor, and a signaling molecule. Sodium lactate challenges can provoke panic in susceptible patients, whereas exercise-associated hyperlactatemia seldom does. However, lactate clamps at rest and during exercise also provide exogenous lactate without reported panic, so endogenous versus exogenous origin cannot explain the observations. We review this three-way comparison and distinguish psychiatric challenges from physiological clamps in participant selection, formulation, exposure kinetics, nutrition, and symptom ascertainment. No universal blood lactate threshold or minimum infusion duration for panic has been established. Sodium loading, acid–base changes, and stereoisomer composition deserve controls; glucose coadministration provides direct evidence that formulation can modify challenge sensitivity. We propose that host vulnerability and the delivered physiological perturbation interact with expectations and perceived control through interoceptive processing. Exercise-related motor prediction is one possible moderator, rather than a necessary condition for tolerating lactate. Insular context gating remains unproven and must be tested against chemical and respiratory explanations in matched-exposure studies. The revised framework accommodates tolerated infusions and occasional exercise-associated panic without treating lactate itself as uniformly threatening or reassuring.

## Introduction

Lactate is an oxidative substrate, a gluconeogenic precursor, and a mediator of metabolic sensing and signaling, rather than merely a waste product (1). In neuronal cell models, lactate regulates differentiation-related gene programs through NDRG3-dependent and independent pathways (2). Those experiments establish signaling potential, not an acute panic mechanism. The lactate–panic question must therefore be framed within contemporary lactate biology.

Panic disorder is characterized by recurrent panic attacks and persistent concern or behavioral change related to future attacks. The interpretation of bodily changes as signals of imminent threat is one proposed contributor to panic vulnerability. Cognitive accounts emphasize catastrophic interpretations of somatic sensations, whereas neurobiological accounts emphasize a distributed fear network linking the insular and prefrontal cortices with the amygdala, hypothalamus, periaqueductal gray, and respiratory brainstem (3, 4). Lactate occupies a distinctive position: it is a circulating metabolite, a cerebral energy substrate and signal, and a long-standing laboratory inducer of panic.

Intravenous sodium lactate was first reported to provoke marked anxiety in susceptible patients (5). Subsequent studies characterized clinical attacks and cerebral blood flow responses (6, 7). An early PET study reported regional activation (8), but its temporopolar interpretation must be qualified by the later correction concerning extracerebral muscle activity (9). Magnetic resonance spectroscopy identified brain lactate responses to infusion, alkalosis, or neural activation in panic disorder (10–13). These findings link vulnerability to lactate-related physiology without establishing concentration as a sufficient cause (14).

The relevant comparison includes endogenous lactate during exercise, exogenous lactate during resting challenges, and exogenous lactate clamped during rest or exercise. This Mini Review examines how exposure, participant characteristics, and context could explain their divergent outcomes. We retain an interoceptive hypothesis but do not assume that infused lactate is intrinsically unexpected or that endogenous lactate is protective. **Table 1** summarizes the three physiological contexts and their key methodological differences.

**Table 1:** Three physiological contexts relevant to the lactate–panic question.

Feature	Exercise alone	Psychiatric challenge at rest	Physiological clamp at rest or exercise
Lactate delivery	Endogenous; workload dependent	Externally imposed, commonly rapid	Externally imposed, adjusted to target
Accompanying signals	Motor effort and cardiorespiratory changes	Formulation, respiratory change, and anticipation	Formulation plus resting or exertional physiology
Motor prediction	Present; may aid predictability	Absent; not necessarily unanticipated	Absent at rest; present during exercise
Population and outcome	Panic disorder studied; occasional attacks	Susceptible patients often panic; controls less often	Small healthy male cohorts; panic not reported or formally diagnosed
Interpretation	Exercise context may moderate response	Susceptibility and protocol must be separated	Exogenous origin does not determine emotional outcome

These are protocol-dependent contrasts, not a matched causal experiment. Tolerance in healthy men cannot establish tolerance in panic disorder; absence of reported panic in metabolic studies is not a prospectively ascertained zero-attack rate.

## The lactate–panic paradox

In a pivotal study of patients with panic disorder, supramaximal exercise produced a mean blood lactate concentration of approximately 10.7 mmol/L, yet only one of 24 participants experienced a panic attack (15). By contrast, historical sodium lactate challenges reported much higher panic rates (5–8); the exercise study noted that its lactate levels exceeded those usually achieved during infusion (15). In healthy volunteers, acute exercise-induced hyperlactatemia did not potentiate a subsequent 35% carbon dioxide challenge (16). Acute aerobic exercise reduced experimentally induced panic in healthy participants (17), attenuated carbon dioxide-evoked panic in patients with panic disorder (18), and reduced cholecystokinin-4-induced panic in both patients and controls (19). These studies are generally small, but their direction of effect is difficult to reconcile with lactate as a sufficient panicogen.

Intervention studies are consistent with this pattern, although they address exercise rather than lactate in isolation. Exercise can reduce anxiety sensitivity (20), has been examined as an adjunct to exposure therapy (21), and exercise avoidance associated with fear of cardiorespiratory sensations has been documented (22). In a recent assessor-blinded randomized trial, 12 weeks of brief, intense exercise used as interoceptive exposure reduced panic severity more than relaxation training, and the benefit persisted during follow-up (23). The trial does not identify lactate as the active mechanism, but it shows the clinical relevance of repeated exposure to intense exercise-related bodily signals.

These observations do not establish a source-specific effect. Exercise protocols differ in fitness, workload, expectations, and symptom assessment, while challenge protocols differ in formulation, rate, and diagnostic criteria. Critically, physiological lactate clamps contradict a categorical association between exogenous exposure and panic.

Miller et al. clamped lactate at approximately 4 mmol/L during 90 minutes of rest followed by 90 minutes of exercise in six healthy active men, separating lactate availability from workload and showing increased lactate oxidation and glucose sparing (24). A distinct nine-man training study used resting targets of 3.5–4.0 mmol/L and exercise-matched concentrations; perceived exertion did not increase with the clamp (25). Neither paper reported panic, although neither used a dedicated panic diagnostic protocol.

In six healthy men, a further study documented approximately 4 mmol/L at rest and 4–7 mmol/L during exercise, volume shifts, and mild systemic alkalosis without reported panic (26). Thus alkalosis and lack of exercise-related motor prediction are not sufficient to make an infusion panicogenic. A contemporary resting study in eight healthy men used racemic sodium lactate for six hours, reaching 2.7 ± 0.5 mmol/L postabsorptively, without reporting panic (27). These healthy cohorts do not establish the response of susceptible patients to matched clamps.

## Lactate exposure and the accompanying physiological event

Circulating lactate supports brain metabolism during exercise (28) and during resting infusion (29). Exercise increases brain lactate signals (30, 31) and cortical glutamate and gamma-aminobutyric acid; sensorimotor gamma-aminobutyric acid changes correlate with blood lactate (32, 33). These associations do not demonstrate an antipanic mechanism or conversion of circulating lactate into inhibitory neurotransmitter. Cerebral uptake depends substantially on arterial concentration (34), with monocarboxylate transporters mediating transport (35). Exogenous origin therefore does not preclude cerebral utilization.

Sodium and acid–base effects require separate consideration. In a randomized human study, sodium lactate and 3% sodium chloride produced similar panic incidence and sodium increases despite alkalosis with lactate and mild acidosis with chloride (36). Lactate was therefore unnecessary under those conditions, but sodium and osmolality were not independently isolated. Sodium-containing solutions, unlike iso-osmolar mannitol, evoked panic-like responses in susceptible rats (37); angiotensin signaling contributed (38). These findings and broader chemosensory work (39) do not establish sodium as the explanation of all human attacks.

Whether unneutralized lactic acid provokes human panic as effectively as equimolar sodium lactate remains unknown; we identified no direct comparison in panic disorder. Equal lactate doses impose different proton, sodium, and osmotic loads. The clamp recipes containing L-lactic acid neutralized with NaOH to pH 4.8 are mixed sodium-lactate/lactic-acid formulations, not sodium-free lactic acid challenges (24–26). Sodium bicarbonate also induced panic without a significant attack-rate difference from sodium lactate in a small human comparison (40), which does not establish equal potency. Rat slice findings that sodium lactate, but not lactic acid, increased relevant neuronal firing (37) cannot answer comparative human panicogenicity.

Several neural mechanisms remain plausible. Lactate–proton transport and buffering may engage acid-sensitive pathways; amygdalar ASIC1A contributes to carbon dioxide-evoked fear in mice (41, 42). HCAR1 mediates exercise-related vascular adaptation in mice (43) and can acutely reduce neuronal activity in rodent and human brain tissue (44). These findings concern different timescales and endpoints. None demonstrates that motor context, receptor signaling, or acid sensing explains the clinical comparisons.

## Factors that may modify challenge susceptibility

### Administration site

Clamps and psychiatric challenges both use peripheral arm veins: the Miller protocol infused through a forearm vein, whereas Coplan et al. used a right antecubital venous line (24, 45). Heated hand venous or arterial catheters were sampling routes, not alternative infusion routes. We identified no randomized arm-versus-other-site panic comparison. Local irritation, dilution, and venous flow may affect the sensation and delivery profile, but infusion site cannot currently explain the contrast.

### Infusate osmolality

A 0.5 mol/L fully dissociated sodium lactate solution has a calculated ideal osmolarity of approximately 1000 mOsm/L; 3% NaCl is approximately 1027 mOsm/L (2 × 30/58.44 mol/L). These estimates are not measured osmolality in mOsm/kg. The clamp mixtures were also hypertonic, and the 2005 study co-infused saline to limit local irritation (26). Final measured infusate osmolality is not consistently reported. Hypertonicity remains a candidate moderator, not a demonstrated universal cause.

### Blood level and infusion duration

The usual challenge uses 0.5 mol/L solution at 10 mL/kg over 20 minutes, equivalent to 5 mmol/kg total lactate if completed (36). This describes dose and protocol duration, not a required blood threshold or latency. Clamps produced overlapping concentrations for much longer without reported panic (**Table 2**); high exercise lactate also seldom provoked attacks. Nonpanicking patients still developed substantial lactate, pyruvate, and pH changes (46). Concentration trajectory, rate, cumulative sodium, and time to attack require joint analysis; early stopping at panic creates outcome-dependent exposure.

**Table 2:** Representative human protocols and limits of the comparison.

Study and participants	Formulation and delivery	Exposure and nutrition	Panic evidence and limitation
Miller et al., 2002, J Physiol (24) 6 healthy active men	L(+)-lactic acid + NaOH; final pH 4.8; forearm venous infusion	90 min rest + 90 min exercise; rest ≈4 mmol/L; exercise target matched 65% peak oxygen uptake; exercise 12 h after standardized snack	No panic reported; metabolic and exertion measures, not formal panic adjudication
Miller et al., 2002, AJP (25) 9 healthy sedentary men, before and after training	L(+)-lactic acid + NaOH; final pH 4.8; peripheral arm infusion	90 min rest + 90 min exercise; rest 3.5–4.0 mmol/L; exercise ≈3.5–4.5 mmol/L; rest 10.5 h and exercise 12 h postfeeding	No panic reported; perceived exertion unchanged with clamp; healthy cohort
Miller et al., 2005 (26) 6 healthy active men	L(+)-lactic acid + NaOH; final pH 4.8; hypertonic infusion with isotonic saline; volume-matched control	90 min rest + 90 min exercise; rest ≈4 and exercise 4–7 mmol/L; nutritional state not used to test panic susceptibility	No panic reported; mild alkalosis and volume shifts; no dedicated panic outcome
Peskind et al., 1998 (36) 8 patients; healthy control group	0.5 mol/L sodium lactate, 3% NaCl, or normal saline; randomized, double-blind IV challenges	Equal-volume 20 min infusions; reference protocol 10 mL/kg; achieved concentrations do not define an attack threshold	Lactate and hypertonic saline had similar panic incidence in patients; sodium and osmolality not independently isolated
Pedersen et al., 2024 (27) 8 healthy men	0.5 mol/L racemic sodium lactate; cubital vein; sodium-matched NaCl control	50 µmol/kg/min for 30 min then 25 for 330 min; postabsorptive lactate 2.7 ± 0.5 mmol/L; overnight 10 h fast; glucose/insulin clamp during final 3 h	No panic reported; metabolic trial with no dedicated panic assessment; D/L and L-only exposure are not interchangeable
Martinsen et al., 1998 (15) 24 patients with panic disorder	Supramaximal exercise; endogenous lactate	Mean blood lactate ≈10.7 mmol/L; no exogenous infusate	1/24 panicked; high concentration is not a sufficient panic trigger; protocol differs from clamps

Infusate concentration is not circulating concentration. Values use each study’s reported blood/plasma measures and are not assumed to be assay-equivalent. IV, intravenous. No panic reported means that the publication did not report an attack, not that a prespecified panic endpoint was negative. No pooled attack-rate comparison is justified across these protocols.

### Stereoisomers

The Miller clamps used L(+)-lactate formulations, whereas historical psychiatric challenges frequently used racemic D,L-lactate (6, 47). In a 19-patient pilot, pure D-lactate induced panic in approximately half, with hypocapnia and alkalosis (47). This weakens an obligatory rapid L-lactate-oxidation explanation but does not identify D-lactate as the sole cause. The tolerated 2024 metabolic infusion was also racemic (27). Formulation and assays must specify separate D and L exposure; routine L-selective measurements do not quantify total racemic lactate.

### Feeding and carbohydrate availability

The Miller studies used standardized meals and overnight postabsorptive conditions, not fed participants who avoided panic solely because they had eaten (24, 25). Feeding can alter baseline substrate availability, so meal timing and composition should be controlled. More directly, a randomized double-blind crossover study in 20 patients and 14 controls found less panic with lactate in 5% dextrose than with lactate in saline; no controls panicked (48). This supports a formulation/nutritional contribution, but does not equate intravenous glucose with a meal or isolate glucose from all accompanying chemical differences.

### Biological sex

Male-only clamp cohorts cannot determine sex effects. In Coplan et al.’s 214-person challenge sample, sex did not add predictive information, although women had different baseline blood gas measures and relatively few female controls limited inference (45). This is evidence against treating sex alone as an established explanation, not proof of equivalent susceptibility. Future studies should prespecify sex effects and record hormonal status where relevant.

### Other host and protocol factors

Diagnosis, current illness and treatment, fitness, anticipatory anxiety, prior challenge experience, and formal panic definitions differ across studies. Coplan et al. linked prelactate fear and the combination of high cortisol and low carbon dioxide to subsequent attacks (45). Baseline hyperventilation, medication exposure or withdrawal, glucose, sodium, ionized calcium, phosphate, renal/hepatic clearance, and cerebral perfusion should therefore be measured or controlled. Associations with these factors do not establish that a single one mediates context gating.

## An interoceptive context-gating model

Interoception encompasses the sensing, integration, and interpretation of signals from within the body. Visceral signals are represented along the insular cortex, with the anterior insula integrating bodily state with attention, emotion, context, and action (49–51). In anxiety, the central disturbance may be less an inaccurate heartbeat count than the excessive precision assigned to bodily prediction errors that could signal threat (52, 53). Predictive-coding accounts describe perception as an interaction among sensory input, prior expectations, and estimates of reliability. Applied to interoception, this account explains how an identical physiological change may be read as ordinary exertion in one setting and evidence of crisis in another (54–56).

We propose that physiological perturbation and host susceptibility interact with expectations and perceived control. During exercise, motor commands, proprioception, and pacing may make bodily arousal predictable. During a well-explained resting clamp, procedural expectations and control could also reduce threat without motor prediction. Conversely, panic-prone participants may interpret challenge-associated dyspnea or dizziness catastrophically. These are testable contextual moderators, not explanations already demonstrated by the clamp studies.

Insular participation is plausible because anterior-insula lesions impair respiratory interoceptive attention (57), and cardiac attention and anxiety recruit insular regions (58). **Figure 1** places this proposed computation downstream of measured exposure and host factors. No cited study demonstrates differential insular gating across all three lactate conditions.

**Figure 1:**
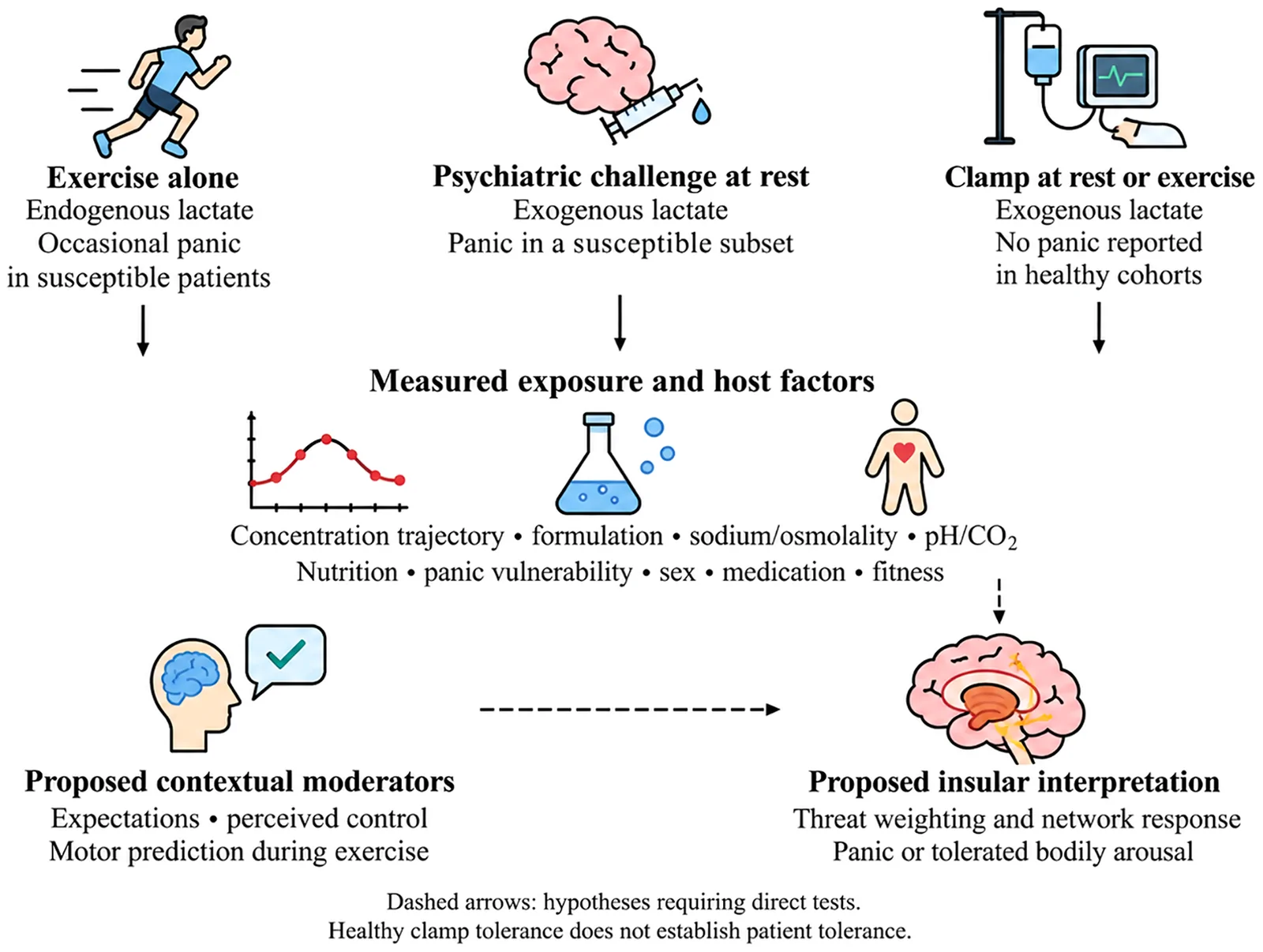
Conditional interoceptive context-gating hypothesis. Three exposure settings feed into measured physiological variables and host vulnerability. Expectations and perceived control are proposed moderators of insular interpretation, rather than established mechanisms. Tolerated clamps at rest are a boundary condition: absence of voluntary motor prediction cannot by itself predict panic. The diagram depicts hypotheses and study constraints, not experimentally established pathways.

The model does not imply that neurons identify lactate’s molecular origin. Transport, metabolism, sodium, pH, and respiratory changes influence bodily evidence; beliefs and context may influence its interpretation. Tolerated resting infusion rules out motor prediction as a necessary condition for avoiding panic. Illness-related endogenous lactate need not have the meaning of voluntary exercise.

## Discussion

The evidence narrows the paradox without resolving its mechanism. Blood lactate elevation, exogenous administration, fasting, mild alkalosis, and absence of exercise are each insufficient as universal explanations. Human sodium-control and glucose-coadministration findings support formulation-related effects (36, 48); anticipatory fear and respiratory state predict challenge outcome (45). The independent contributions of rate, stereoisomers, sex, and perceived control remain unresolved. These variables should be tested jointly, rather than assigning the contrast to source by exclusion.

Related frameworks should be interpreted within their scope. Kong’s “lactate window” hypothesis concerns exposure and recovery dynamics in depression (59), while his adolescent review discusses contextual responses to exercise-related arousal (60). Aldisi et al. assessed gastric interoception in women with eating disorders (61); Chen associated depression exercise outcomes with adherence and social support (62). These papers motivate measurement of relevant signals and context but provide no direct evidence that lactate causes or prevents acute panic.

Sepsis remains a separate boundary condition. We identified no prospective study pairing acute panic assessment and serial lactate in septic patients with pre-existing panic disorder. A small tracer study linked hyperlactatemia mainly to production rather than reduced clearance (63), but this is not universal or equivalent to exercise. Infection, respiratory or circulatory disturbances, and treatment alter the physiological event. Delirium and sedation complicate assessment (64); tachycardia, tachypnea, or post-illness anxiety cannot establish acute panic.

Unexpected illness might enhance threat interpretation despite endogenous lactate production, but this remains hypothetical. Overlapping lactate elevations in psychogenic hyperventilation and suspected urinary sepsis occurred with different pH and carbon dioxide profiles (65); that comparison did not test panic susceptibility during sepsis. A prospective observational study would require prior panic diagnosis, independently adjudicated acute attacks, serial lactate and blood gases, delirium assessment, and illness severity and treatment covariates. Neither undocumented panic nor nonspecific anxiety answers the question.

## Pyruvate as a comparator

Pyruvate was measured alongside lactate in psychiatric challenge studies (46), but measurement is not administration. Human pyruvate infusion studies exist: Hermann et al. gave two 15-minute intracoronary infusions to eight patients with dilated cardiomyopathy and observed hemodynamic effects (66). That study assessed neither panic disorder nor a matched peripheral lactate challenge. We identified no controlled human panic study comparing pyruvate alone or combined with lactate against lactate alone. A future comparator could test substrate/redox specificity only with matched delivery and sodium, pH, osmotic, and respiratory controls.

A decisive study would compare exercise alone, L-lactate clamp at rest, and the same clamp during exercise within participants with panic disorder and matched controls. It should match concentration trajectories and exposure area, characterize formulation and nutrition, and prespecify validated panic outcomes. Arterial or consistently arterialized sampling, separate D/L assays when needed, blood gases, sodium, measured osmolality, glucose, and ventilation are essential. Expectations and perceived control should be measured rather than inferred from assigned condition; appropriate imaging could test insular responses.

Chemical controls should include sodium chloride and bicarbonate, with separate osmotic controls where appropriate; none matches every chemical and respiratory variable. Subsequent randomized contextual manipulations at fixed exposure could test whether predictability or control changes panic and insular responses. Neuromuscular stimulation could further separate muscular lactate production from voluntary action. A context effect persisting after chemical and respiratory adjustment would support gating; explanation by these physiological variables alone, with no contextual effect, would weaken it. Acid or pyruvate challenges require separate formulation and safety evaluation.

Limitations include small samples, male-only physiological cohorts, heterogeneous assays and panic definitions, and no matched three-condition imaging study in panic disorder. Metabolic studies did not prospectively ascertain syndromal panic. Proton spectroscopy cannot distinguish circulating from locally produced lactate, and cortical neurotransmitter associations and animal chemosensory findings do not establish human insular causality. Direct lactic-acid comparisons, pyruvate panic challenges, and sepsis-specific evidence remain unavailable in the literature identified.

Lactate exposure is neither uniformly panicogenic nor uniformly protective. Existing controls exclude several simple universal explanations while supporting contributions from formulation and host state. Conditional context gating is a falsifiable hypothesis within that evidence, not a completed resolution of the paradox.

## References

[1] Brooks GA Arevalo JA Osmond AD Leija RG Curl CC Tovar AP . Lactate in contemporary biology: a phoenix risen. J Physiol. (2022) 600:1229–51. doi: 10.1113/JP280955 33566386 PMC9188361

[2] Xu Y Kusuyama J Osana S Matsuhashi S Li L Takada H . Lactate promotes neuronal differentiation of SH-SY5Y cells by lactate-responsive gene sets through NDRG3-dependent and -independent manners. J Biol Chem. (2023) 299:104802. doi: 10.1016/j.jbc.2023.104802 37172727 PMC10276297

[3] Clark DM . A cognitive approach to panic. Behav Res Ther. (1986) 24:461–70. doi: 10.1016/0005-7967(86)90011-2 3741311

[4] Gorman JM Kent JM Sullivan GM Coplan JD . Neuroanatomical hypothesis of panic disorder, revised. Am J Psychiatry. (2000) 157:493–505. doi: 10.1176/appi.ajp.157.4.493 10739407

[5] Pitts FN Jr McClure JN Jr . Lactate metabolism in anxiety neurosis. N Engl J Med. (1967) 277:1329–36. doi: 10.1056/NEJM196712212772502 6081131

[6] Liebowitz MR Fyer AJ Gorman JM Dillon D Appleby IL Levy G . Lactate provocation of panic attacks. I. Clinical and behavioral findings. Arch Gen Psychiatry. (1984) 41:764–70. doi: 10.1001/archpsyc.1984.01790190038004 6742978

[7] Stewart RS Devous MD Sr Rush AJ Lane L Bonte FJ . Cerebral blood flow changes during sodium-lactate-induced panic attacks. Am J Psychiatry. (1988) 145:442–9. doi: 10.1176/ajp.145.4.442 3258132

[8] Reiman EM Raichle ME Robins E Mintun MA Fusselman MJ Fox PT . Neuroanatomical correlates of a lactate-induced anxiety attack. Arch Gen Psychiatry. (1989) 46:493–500. doi: 10.1001/archpsyc.1989.01810060013003 2786401

[9] Drevets WC Videen TQ MacLeod AK Haller JW Raichle ME . PET images of blood flow changes during anxiety: correction. Science. (1992) 256:1696. doi: 10.1126/science.256.5064.1696 1609283

[10] Dager SR Marro KI Richards TL Metzger GD . Preliminary application of magnetic resonance spectroscopy to investigate lactate-induced panic. Am J Psychiatry. (1994) 151:57–63. doi: 10.1176/ajp.151.1.57 8267135

[11] Maddock RJ . The lactic acid response to alkalosis in panic disorder: an integrative review. J Neuropsychiatry Clin Neurosci. (2001) 13:22–34. doi: 10.1176/jnp.13.1.22 11207326

[12] Maddock RJ Buonocore MH Copeland LE Richards AL . Elevated brain lactate responses to neural activation in panic disorder: a dynamic 1H-MRS study. Mol Psychiatry. (2009) 14:537–45. doi: 10.1038/sj.mp.4002137 18180759

[13] Maddock RJ Buonocore MH Miller AR Yoon JH Soosman SK Unruh AM . Abnormal activity-dependent brain lactate and glutamate+glutamine responses in panic disorder. Biol Psychiatry. (2013) 73:1111–9. doi: 10.1016/j.biopsych.2012.12.015 23332354 PMC3636170

[14] Riske L Thomas RK Baker GB Dursun SM . Lactate in the brain: an update on its relevance to brain energy, neurons, glia and panic disorder. Ther Adv Psychopharmacol. (2017) 7:85–9. doi: 10.1177/2045125316675579 28255438 PMC5315230

[15] Martinsen EW Raglin JS Hoffart A Friis S . Tolerance to intensive exercise and high levels of lactate in panic disorder. J Anxiety Disord. (1998) 12:333–42. doi: 10.1016/S0887-6185(98)00018-8 9699117

[16] Esquivel G Schruers K Kuipers H Griez E . The effects of acute exercise and high lactate levels on 35% CO2 challenge in healthy volunteers. Acta Psychiatr Scand. (2002) 106:394–7. doi: 10.1034/j.1600-0447.2002.01333.x 12366475

[17] Ströhle A Feller C Onken M Godemann F Heinz A Dimeo F . The acute antipanic activity of aerobic exercise. Am J Psychiatry. (2005) 162:2376–8. doi: 10.1176/appi.ajp.162.12.2376 16330604

[18] Esquivel G Díaz-Galvis J Schruers K Berlanga C Lara-Muñoz C Griez E . Acute exercise reduces the effects of a 35% CO2 challenge in patients with panic disorder. J Affect Disord. (2008) 107:217–20. doi: 10.1016/j.jad.2007.07.022 17727960

[19] Ströhle A Graetz B Scheel M Wittmann A Feller C Heinz A . The acute antipanic and anxiolytic activity of aerobic exercise in patients with panic disorder and healthy control subjects. J Psychiatr Res. (2009) 43:1013–7. doi: 10.1016/j.jpsychires.2009.02.004 19289240

[20] Smits JAJ Berry AC Rosenfield D Powers MB Behar E Otto MW . Reducing anxiety sensitivity with exercise. Depress Anxiety. (2008) 25:689–99. doi: 10.1002/da.20411 18729145

[21] Bischoff S Wieder G Einsle F Petzold MB Janßen C Mumm JLM . Running for extinction? Aerobic exercise as an augmentation of exposure therapy in panic disorder with agoraphobia. J Psychiatr Res. (2018) 101:34–41. doi: 10.1016/j.jpsychires.2018.03.001 29539585

[22] Muotri RW Bernik MA . Panic disorder and exercise avoidance. Braz J Psychiatry. (2014) 36:68–75. doi: 10.1590/1516-4446-2012-1012 24604463

[23] Muotri RW Luciano AC Garrudo Guirado A Lotufo Neto F Bernik M . Brief intermittent intense exercise as interoceptive exposure for panic disorder: a randomized controlled clinical trial. Front Psychiatry. (2026) 16:1739639. doi: 10.3389/fpsyt.2025.1739639 41737725 PMC12926410

[24] Miller BF Fattor JA Jacobs KA Horning MA Navazio F Lindinger MI . Lactate and glucose interactions during rest and exercise in men: effect of exogenous lactate infusion. J Physiol. (2002) 544:963–75. doi: 10.1113/jphysiol.2002.027128 12411539 PMC2290635

[25] Miller BF Fattor JA Jacobs KA Horning MA Suh SH Navazio F . Metabolic and cardiorespiratory responses to “the lactate clamp”. Am J Physiol Endocrinol Metab. (2002) 283:E889–98. doi: 10.1152/ajpendo.00266.2002 12376315

[26] Miller BF Lindinger MI Fattor JA Jacobs KA LeBlanc PJ Duong M . Hematological and acid-base changes in men during prolonged exercise with and without sodium-lactate infusion. J Appl Physiol. (2005) 98:856–65. doi: 10.1152/japplphysiol.00753.2004 15475600

[27] Pedersen MGB Rittig N Bangshaab M Berg-Hansen K Gopalasingam N Gormsen LC . Effects of exogenous lactate on lipid, protein, and glucose metabolism—a randomized crossover trial in healthy males. Am J Physiol Endocrinol Metab. (2024) 326:E443–53. doi: 10.1152/ajpendo.00301.2023 38324259 PMC11193511

[28] van Hall G Strømstad M Rasmussen P Jans O Zaar M Gam C . Blood lactate is an important energy source for the human brain. J Cereb Blood Flow Metab. (2009) 29:1121–9. doi: 10.1038/jcbfm.2009.35 19337275

[29] Boumezbeur F Petersen KF Cline GW Mason GF Behar KL Shulman GI . The contribution of blood lactate to brain energy metabolism in humans measured by dynamic 13C nuclear magnetic resonance spectroscopy. J Neurosci. (2010) 30:13983–91. doi: 10.1523/JNEUROSCI.2040-10.2010 20962220 PMC2996729

[30] Maddock RJ Casazza GA Buonocore MH Tanase C . Vigorous exercise increases brain lactate and Glx (glutamate+glutamine): a dynamic 1H-MRS study. NeuroImage. (2011) 57:1324–30. doi: 10.1016/j.neuroimage.2011.05.048 21640838

[31] Dennis A Thomas AG Rawlings NB Near J Nichols TE Clare S . An ultra-high field magnetic resonance spectroscopy study of post exercise lactate, glutamate and glutamine change in the human brain. Front Physiol. (2015) 6:351. doi: 10.3389/fphys.2015.00351 26732236 PMC4681779

[32] Maddock RJ Casazza GA Fernandez DH Maddock MI . Acute modulation of cortical glutamate and GABA content by physical activity. J Neurosci. (2016) 36:2449–57. doi: 10.1523/JNEUROSCI.3455-15.2016 26911692 PMC6705493

[33] Coxon JP Cash RFH Hendrikse JJ Rogasch NC Stavrinos E Suo C . GABA concentration in sensorimotor cortex following high-intensity exercise and relationship to lactate levels. J Physiol. (2018) 596:691–702. doi: 10.1113/JP274660 29159914 PMC5813602

[34] Siebenmann C Sørensen H Bonne TC Zaar M Aachmann-Andersen NJ Nordsborg NB . Cerebral lactate uptake during exercise is driven by the increased arterial lactate concentration. J Appl Physiol. (2021) 131:1824–30. doi: 10.1152/japplphysiol.00505.2021 34734784

[35] Bergersen LH . Lactate transport and signaling in the brain: potential therapeutic targets and roles in body–brain interaction. J Cereb Blood Flow Metab. (2015) 35:176–85. doi: 10.1038/jcbfm.2014.206 25425080 PMC4426752

[36] Peskind ER Jensen CF Pascualy M Tsuang D Cowley D Martin DC . Sodium lactate and hypertonic sodium chloride induce equivalent panic incidence, panic symptoms, and hypernatremia in panic disorder. Biol Psychiatry. (1998) 44:1007–16. doi: 10.1016/S0006-3223(98)00053-5 9821565

[37] Molosh AI Johnson PL Fitz SD DiMicco JA Herman JP Shekhar A . Changes in central sodium and not osmolarity or lactate induce panic-like responses in a model of panic disorder. Neuropsychopharmacology. (2010) 35:1333–47. doi: 10.1038/npp.2010.2 20130534 PMC2855744

[38] Johnson PL Sajdyk TJ Fitz SD Hale MW Lowry CA Hay-Schmidt A . Angiotensin II's role in sodium lactate-induced panic-like responses in rats with repeated urocortin 1 injections into the basolateral amygdala: amygdalar angiotensin receptors and panic. Prog Neuro Psychopharmacol Biol Psychiatry. (2013) 44:248–56. doi: 10.1016/j.pnpbp.2013.02.014 23523745 PMC3665353

[39] Vollmer LL Strawn JR Sah R . Acid–base dysregulation and chemosensory mechanisms in panic disorder: a translational update. Transl Psychiatry. (2015) 5:e572. doi: 10.1038/tp.2015.67 26080089 PMC4471296

[40] Gorman JM Battista D Goetz RR Dillon DJ Liebowitz MR Fyer AJ . A comparison of sodium bicarbonate and sodium lactate infusion in the induction of panic attacks. Arch Gen Psychiatry. (1989) 46:145–50. doi: 10.1001/archpsyc.1989.01810020047008 2536538

[41] Ziemann AE Allen JE Dahdaleh NS Drebot II Coryell MW Wunsch AM . The amygdala is a chemosensor that detects carbon dioxide and acidosis to elicit fear behavior. Cell. (2009) 139:1012–21. doi: 10.1016/j.cell.2009.10.029 19945383 PMC2808123

[42] Quagliato LA Freire RC Nardi AE . The role of acid-sensitive ion channels in panic disorder: a systematic review of animal studies and meta-analysis of human studies. Transl Psychiatry. (2018) 8:185. doi: 10.1038/s41398-018-0238-z 30194289 PMC6128878

[43] Morland C Andersson KA Haugen ØP Hadzic A Kleppa L Gille A . Exercise induces cerebral VEGF and angiogenesis via the lactate receptor HCAR1. Nat Commun. (2017) 8:15557. doi: 10.1038/ncomms15557 28534495 PMC5457513

[44] Briquet M Rocher AB Alessandri M Rosenberg N de Castro Abrantes H Wellbourne-Wood J . Activation of lactate receptor HCAR1 down-modulates neuronal activity in rodent and human brain tissue. J Cereb Blood Flow Metab. (2022) 42:1650–65. doi: 10.1177/0271678X221080324 35240875 PMC9441721

[45] Coplan JD Goetz R Klein DF Papp LA Fyer AJ Liebowitz MR . Plasma cortisol concentrations preceding lactate-induced panic. Psychological, biochemical, and physiological correlates. Arch Gen Psychiatry. (1998) 55:130–6. doi: 10.1001/archpsyc.55.2.130 9477926

[46] Liebowitz MR Gorman JM Fyer AJ Levitt M Dillon D Levy G . Lactate provocation of panic attacks. II. Biochemical and physiological findings. Arch Gen Psychiatry. (1985) 42:709–19. doi: 10.1001/archpsyc.1985.01790300077010 4015313

[47] Gorman JM Goetz RR Dillon D Liebowitz MR Fyer AJ Davies S . Sodium D-lactate infusion of panic disorder patients. Neuropsychopharmacology. (1990) 3:181–9. 2114119

[48] George DT Lindquist T Nutt DJ Ragan PW Alim T McFarlane V . Effect of chloride or glucose on the incidence of lactate-induced panic attacks. Am J Psychiatry. (1995) 152:692–7. doi: 10.1176/ajp.152.5.692 7726308

[49] Craig AD . How do you feel? Interoception: the sense of the physiological condition of the body. Nat Rev Neurosci. (2002) 3:655–66. doi: 10.1038/nrn894 12154366

[50] Craig AD . How do you feel—now? The anterior insula and human awareness. Nat Rev Neurosci. (2009) 10:59–70. doi: 10.1038/nrn2555 19096369

[51] Critchley HD Wiens S Rotshtein P Öhman A Dolan RJ . Neural systems supporting interoceptive awareness. Nat Neurosci. (2004) 7:189–95. doi: 10.1038/nn1176 14730305

[52] Paulus MP Stein MB . An insular view of anxiety. Biol Psychiatry. (2006) 60:383–7. doi: 10.1016/j.biopsych.2006.03.042 16780813

[53] Paulus MP Stein MB . Interoception in anxiety and depression. Brain Struct Funct. (2010) 214:451–63. doi: 10.1007/s00429-010-0258-9 20490545 PMC2886901

[54] Seth AK . Interoceptive inference, emotion, and the embodied self. Trends Cognit Sci. (2013) 17:565–73. doi: 10.1016/j.tics.2013.09.007 24126130

[55] Barrett LF Simmons WK . Interoceptive predictions in the brain. Nat Rev Neurosci. (2015) 16:419–29. doi: 10.1038/nrn3950 26016744 PMC4731102

[56] Khalsa SS Adolphs R Cameron OG Critchley HD Davenport PW Feinstein JS . Interoception and mental health: a roadmap. Biol Psychiatry Cognit Neurosci Neuroimaging. (2018) 3:501–13. doi: 10.1016/j.bpsc.2017.12.004 29884281 PMC6054486

[57] Wang X Wu Q Egan L Gu X Liu P Gu H . Anterior insular cortex plays a critical role in interoceptive attention. eLife. (2019) 8:e42265. doi: 10.7554/eLife.42265 30985277 PMC6488299

[58] Tan Y Wei D Zhang M Yang J Jelinčić V Qiu J . The role of mid-insula in the relationship between cardiac interoceptive attention and anxiety: evidence from an fMRI study. Sci Rep. (2018) 8:17280. doi: 10.1038/s41598-018-35635-6 30467392 PMC6250688

[59] Kong J . The “lactate window” hypothesis: exercise-induced lactate dynamics in neurometabolic remodeling and symptom-dimensional exercise prescription for depression. Explor Neurosci. (2026) 5:1006142. doi: 10.37349/en.2026.1006142

[60] Kong J . Psychophysiological pathways linking physical activity and mental health in adolescents: a narrative review of autonomic regulation, hypothalamic–pituitary–adrenal axis function, neuroplasticity, and sleep rhythms. Lifespan Dev Ment Health. (2026) 2:10014. doi: 10.70322/ldmh.2026.1001439040700

[61] Aldisi D Alyami SH Alhamdan A Alebrahim HM Almadani AH Abulmeaty MMA . Association of dietary intake with interoception and gastric myoelectric activity in women with binge eating disorder and bulimia nervosa. Actas Esp Psiquiatri. (2024) 52:777–85. doi: 10.62641/aep.v52i6.1734 39665608 PMC11636537

[62] Chen GR . Influencing factors on the efficacy of aerobic exercise therapy in depression: a single-centre cross-sectional study. Actas Esp Psiquiatri. (2024) 52:107–13. doi: 10.62641/aep.v52i2.1585 38622008 PMC11015741

[63] Revelly JP Tappy L Martinez A Bollmann M Cayeux MC Berger MM . Lactate and glucose metabolism in severe sepsis and cardiogenic shock. Crit Care Med. (2005) 33:2235–40. doi: 10.1097/01.CCM.0000181525.99295.8F 16215376

[64] Yamamoto T Mizobata Y Kawazoe Y Miyamoto K Ohta Y Morimoto T . Incidence, risk factors, and outcomes for sepsis-associated delirium in patients with mechanical ventilation: a sub-analysis of a multicenter randomized controlled trial. J Crit Care. (2020) 56:140–4. doi: 10.1016/j.jcrc.2019.12.018 31901649

[65] Urushidani S Kuriyama A Matsumura M . Blood gas analysis results and serum lactate levels in patients with psychogenic hyperventilation and urinary tract infection with suspected sepsis: a retrospective comparative study. Intern Med. (2022) 61:1699–706. doi: 10.2169/internalmedicine.8434-21 34707052 PMC9259313

[66] Hermann HP Pieske B Schwarzmüller E Keul J Just H Hasenfuss G . Haemodynamic effects of intracoronary pyruvate in patients with congestive heart failure: an open study. Lancet. (1999) 353:1321–3. doi: 10.1016/S0140-6736(98)06423-X 10218531

